# Application of metagenomic next-generation sequencing in the clinical diagnosis of infectious diseases after allo-HSCT: a single-center analysis

**DOI:** 10.1186/s12879-024-09153-y

**Published:** 2024-03-04

**Authors:** Hailong Yuan, Xiaolu Ma, Jianli Xu, Peng Han, Guanhua Rao, Gang Chen, Kaile Zhang, Ruixue Yang, Chuixia Han, Ming Jiang

**Affiliations:** 1 Hematology Center, The First Affiliated Hospital of Xinjiang Medical University, Xinjiang Institute of Hematology, No.137 Liyushan South Road, Urumqi, 830054 China; 2 Department of Hematology, Guangyuan Central Hospital, Guangyuan, Sichuan Province China; 3 Genskey Medical Technology Co., Ltd, Beijing, China

**Keywords:** Allogeneic hematopoietic stem cell transplantation, Metagenomic high-throughput sequencing (mNGS), Infection, Non-infection, Fever

## Abstract

**Background:**

We investigated the value of metagenomic next-generation sequencing (mNGS) in diagnosing infectious diseases in patients receiving allogeneic hematopoietic stem cell transplantation (allo-HSCT).

**Methods:**

Fifty-four patients who had fever following allo-HSCT from October 2019 to February 2022 were enrolled. Conventional microbiological tests (CMTs) and mNGS, along with imaging and clinical manifestations, were used to diagnose infection following allo-HSCT. The clinical diagnostic value of mNGS was evaluated.

**Results:**

A total of 61 mNGS tests were performed, resulting in the diagnosis of 46 cases of infectious diseases. Among these cases, there were 22 cases of viral infection, 13 cases of fungal infection, and 11 cases of bacterial infection. Moreover, 27 cases (58.7%) were classified as bloodstream infections, 15 (32.6%) as respiratory infections, 2 (4.3%) as digestive system infections, and 2 (4.3%) as central nervous system infections. Additionally, there were 8 cases with non-infectious diseases (8/54, 14.81%), including 2 cases of interstitial pneumonia, 2 cases of bronchiolitis obliterans, 2 cases of engraftment syndrome, and 2 cases of acute graft-versus-host disease. The positive detection rates of mNGS and CMT were 88.9% and 33.3%, respectively, with significant differences (*P* < 0.001). The sensitivity of mNGS was 97.82%, the specificity was 25%, the positive predictive value was 93.75%, and the negative predictive value was 50%. Following treatment, 51 patients showed improvement, and 3 cases succumbed to multidrug-resistant bacterial infections.

**Conclusions:**

mNGS plays an important role in the early clinical diagnosis of infectious diseases after allo-HSCT, which is not affected by immunosuppression status, empiric antibiotic therapy, and multi-microbial mixed infection.

## Background

Allo-hematopoietic stem cell transplantation (allo-HSCT) is an effective therapeutic approach for many hematologic malignancies as well as bone marrow failure disorders. Infection is still a major concern affecting the outcome of allo-HSCT. According to the guidelines of the European Society for Blood and Marrow Transplantation Working Group on infectious diseases, 22.3% of patients after allo-HSCT would succumb to infections, including 59.4% of unexplained infections [1]. Allo-HSCT pretreatment protocols, reconstitution of the immune system, immunosuppressant administration, and graft-versus-host disease (GVHD) are the main risk factors for infection following allo-HSCT [2, 3]. Targeted treatment based on timely and accurate identification of the cause of infection is important to reduce the mortality of patients receiving allo-HSCT.

Conventional microbiological tests (CMTs), such as smear and culture, are commonly used for infection diagnosis [4]. However, infection with unusual pathogens is usually observed after allo-HSCT, presenting with atypical manifestations, and mixed infection is more prone to occur [1]. Therefore, multiple CMTs are required to identify the causative pathogens or exclude infectious diseases. Although antibody testing is also a major method for diagnosing infectious diseases, patients have difficulty producing antibody responses due to immunocompromised status after allo-HSCT [5]. Identification of the pathogen by biopsy or fine needle aspiration has a high risk of secondary infection and bleeding [6]. Therefore, CMT is still the main method of clinical identification of pathogens. However, there may be a low sensitivity because some pathogens cannot be cultured or have harsh culture conditions, or the pathogens have been inhibited by previously given antibiotics [7, 8], especially in immunocompromised patients [3, 9]. Other detection methods of high sensitivity are needed.

Metagenomic next-generation sequencing (mNGS) is used to facilitate the diagnosis of potential infections through the detection of free DNA in specimens such as plasma, cerebrospinal fluid (CSF), and respiratory secretion. It can be used for detecting pathogens such as bacteria, viruses, and fungi, as well as multiple pathogen infections [10–14]. Compared with CMT, mNGS is faster and more accurate, has higher sensitivity, and can identify a wide range of infections [15]. However, the application of mNGS in diagnosing infectious diseases in patients after allo-HSCT remains limited.

Therefore, in this study, we investigated the clinical diagnostic value of mNGS in infectious diseases in patients following allo-HSCT. Our findings suggest that mNGS testing may be used for the timely clinical diagnosis of complex infections in immunocompromised patients, thus reducing infection-related death.

## Methods

### Patients

Patients with fever of unknown origin following allo-HSCT, who were admitted at the transplant center of the First Affiliated Hospital of Xinjiang Medical University from October 2019 to February 2022, were enrolled. Inclusion criteria: 1) Patients were administrated with immunosuppressants, prophylactic, or empiric anti-infective therapy, and had persistent fever of unknown origin for more than 7 days (axillary temperature > 38 °C) after allo-HSCT. 2) Symptoms of the respiratory tract, digestive tract, and central nervous system were presented, such as cough and sputum, shortness of breath, and dyspnea. 3) Patients with imaging findings of patches, cords, nodules, ground glass shadows, exudation, consolidation, interstitial changes, bronchiectasis, pleural effusion, meningitis, etc. 4) SPO2 (partial pressure of oxygen) ≤ 70 mmHg, FEV1/FVC%≤70%, and FEV1% pred<80%. Exclusion criteria: 1) Cases of non-HSCT transplantation. 2) Cases with controlled infection after prophylactic or empirical anti-infection treatment. 3) Cases lost to follow-up or with incomplete clinical data. The study was performed following the Declaration of Helsinki and approved by the Ethics Review Board of the First Affiliated Hospital of Xinjiang Medical University. Written informed consent was obtained from each enrolled patient.

### Clinical routine examination and imaging examination

Routine examinations included blood routine tests, testings for galactomannan, (1,3)-β-D dextran, pneumonia antibodies, inflammatory factors, cellular immunity, cytokine, humoral immunity, and pulmonary function, and blood gas analysis. Patients received lung CT, abdominal ultrasound examinations, and cranial MRI examinations, and the imaging changes were dynamically followed up.

### CMTs

All 54 patients in the study underwent blood, urine, stool, and secretion cultures. Patients who underwent lumbar puncture also had CSF cultures performed simultaneously. In addition, bronchoalveolar lavage fluid (BALF) was examined for respiratory infections, including exfoliative cytology, bacterial and fungal cultures, tuberculosis cultures, pathogenic smears, Xport, and acid-fast staining smears. CSF samples were sent for biochemical and routine testing, general bacterial smears and cultures, and cytopathological examination. The laboratory employed standard aerobic culture methods to process the clinical specimens, following the protocols established for clinical microbiology laboratories for pathogen detection. Different specimens were inoculated on different agar plates (blood, chocolate, and Maconkey’s agar) based on the sample type.

### Metagenomic next-generation sequencing

For patients with an undetermined pathogen, mNGS was performed on the DNA samples only. The specimens of peripheral blood (5 ml each), BALF (10 ml each), and CSF (2 ml each) were collected and mNGS was performed to detect the potential pathogens, as previously described [16]. The sequencing was performed by Genskey Medical Technology Co., Ltd (Beijing, China). In brief, DNA was generated using the Genskey kit (2053A The sequencing was performed by Genskey Medical Technology Co., Ltd (Beijing, China). In brief, DNA was generated using the Genskey kit (2053A, Genskey, Tianjin, China). The library was constructed with a library construction kit (2102, Genskey, Tianjin, China). The concentration of the library was determined using the Qubit ® 1×dsDNA HS kit (Q33230, Invitrogen, CA). After thermal denaturation, library mixing, and DNB preparation, all libraries were sequenced with single-stranded circular DNA and added by 2–3 quantitative sets to obtain DNA nanospheres. These DNA nanospheres were sequenced using the MGISEQ-200 sequencing platform (MGI, Shenzhen, China).

To ensure the quality of the sequencing data, fastp (v0.22.0) and Komplexity (v0.3.6) were used to filter the raw reads for adapter contamination, low-quality reads, and low-complexity reads. The mapped reads were classified based on the NCBI Taxonomy assignment of the reference genomes. The uniquely mapped reads to a specific species were used for the identification of pathogens. Moreover, microorganisms with well-defined pathogenicity and uncommon colonization were considered pathogenic bacteria, particularly in cases where the number of pathogen reads was low.

According to the previous study [17], we employed certain measures to eliminate false-positive read alignments with closely related taxa. These measures involved quantifying the number of species-specific read assignments and interspecies read assignments (referred to as “unique mapping reads” and “multiple mapping reads” respectively), determining the pairwise sequence identity (referred to as “identity”), and introducing two parameters named MU and TOP. MU represented the ratio between multiple mapping reads and unique mapping reads within a genome, while TOP denoted the ratio of unique mapping reads in a genome to the highest-ranked genome in the same genus. To establish the thresholds for MU and TOP for each species, we utilized virtual metagenomes generated through in silico methods to simulate the conditions of a single-pathogen infection. Five random subspecies were selected, and the average values of MU and TOP were calculated for these subspecies. Any organisms surpassing the thresholds for MU or TOP were considered false positives and subsequently removed from consideration. Additionally, we combined samples of CSF with other less complex bodily fluids, such as blood samples, in the same run to prevent any bleeding within the sequencing process.

In our study, we used dual-index adapters to prevent index bleeding. To assess both within-run and run-to-run sequencing bleeding, we employed negative controls to monitor for any index bleeding and other contaminations, following the methodology described in a previous study [17]. We developed three new negative controls specifically designed to eliminate contaminants from CSF sampling in the mNGS laboratory and the normal human flora. These controls were named “CumulativeBatchNC” (BatchNCs under surveillance for 5 months), “NegativeSamples” (negative results obtained from conventional tests), and “HealthyNC” (samples from healthy individuals). Organisms were considered significant if they were detected at a fold-change greater than 5 in the test samples compared to the controls. Subsequently, a list of organisms (referred to as the PCR-organism list) was utilized to remove any contaminants introduced during PCR amplification. The FASTQ files have been uploaded to the NCBI-SRA database (http://www.ncbi.nlm.nih.gov/bioproject/1025963). Furthermore, despite the high presence of host sequences in the blood samples, we first removed all the host sequences and then analyzed the microbial sequences during bioinformatics analysis. In both blood and CSF samples, microbial sequences were minimal, reducing the risk of microbial sequence bleeding between blood and CSF samples.

### Diagnostic criteria

The clinical diagnosis of infectious diseases was based on diagnostic criteria for infection after HSCT [18, 19]. The clinical diagnosis of non-infectious diseases, such as bronchiolitis obliterans (BOS) and interstitial pneumonia (IP), was made using established criteria as described in previous studies [20, 21].

### Statistical analysis

Statistics analyses were performed by using SPSS 26.0. Comparison between the results of CMTs and mNGS was conducted by using Chi-square for 2 × 2 contingency tables. Agreement analysis was performed using the Kappa test. A higher Cohen’s kappa coefficient indicates greater agreement. According to mNGS report, Scatter plots were generated by Graphpad6.0 based on log10 conversion of sequencing reading and relative abundance of microorganisms.

## Results

### Baseline characteristics of patients

A total of 54 patients after allo-HSCT were enrolled, including 39 males and 15 females, with a median age of 40 (7–68) years. Their baseline characteristics are listed in Table 1. Among 54 patients, there were 27 cases of acute myeloid leukemia, 14 cases of acute lymphoblastic leukemia, 4 cases of chronic myelomonocytic leukemia, 6 cases of myelodysplastic syndrome, and, 3 cases of aplastic anemia.

A total of 61 mNGS tests were performed for 54 patients. According to clinical and microbiological diagnosis, 46 cases (46/54, 85.2%) were determined to have infectious diseases, including 27 cases (58.7%) of bloodstream infections, 15 cases (32.6%) of respiratory infections, 2 cases (4.3%) of digestive system infections, and 2 cases (4.3%) of central nervous system infections.

Out of the 54 patients, eight cases (14.81%) were identified as non-infectious diseases. Specifically, two cases were diagnosed as IP, and two cases as bronchiolitis obliterans (BOS). In one IP patient, a total of 14 pathogens were detected in the BALF samples, including 74,205 reads of *Acinetobacter baumannii*, 211 reads of *Streptococcus pneumoniae*, and 59 reads of *Pseudomonas aeruginosa*. However, this result did not align with the patient’s symptoms, signs, and imaging examination findings, leading to its classification as a colonization event. Epstein-Barr virus (EBV) and torque teno virus (TTV) were detected in the peripheral blood of one IP patient, while TTV and EBV were detected in the peripheral blood of two BOS patients. However, these findings also did not correspond with the patient’s clinical manifestations, indicating colonization rather than infection. As a result, their diagnoses were established through assessment of clinical symptoms, elevated protein factors (such as the soluble form suppression of tumorigenicity 2 and soluble tumor necrosis factor receptor I), lung function, and high-resolution CT scans of the lungs. Among the remaining four cases (two with engraftment syndrome and two with acute GVHD), neither bacterial, fungal, nor viral pathogens of clinical significance were detected through CMT and NGS. Furthermore, no significant imaging abnormalities were observed. In addition to fever, two patients with engraftment syndrome exhibited symptoms such as rash, shortness of breath, and dyspnea, accompanied by significant increases in IL-10 and C-reactive protein levels. Two cases of acute GVHD presented with skin pruritus and diarrhea, along with elevated levels of IL-6, IL-8, and elafin.

Regarding the initial examination samples of the 54 patients, peripheral blood was the most commonly collected (39 cases, 72.2%), followed by BALF (13 cases, 24.1%), and CSF (2 cases, 3.7%).

**Table 1:** General information of patients

	Patients with allo-HSCT (*n* = 54)
Age (years)	40 (7–68)
Sex (male/female)	39/15
Temperature (°C)	38.45 (36.5–40.7)
Laboratory tests	
CRP (mg/L)	51.8 (4-528)
IL-6 (pg/mL)	21.6 (0.08–5001)
PCT (µg/L)	0.11 (0.01–80.41)
WBC (×10^9^/L)	4.43 (0.01–19.60)
Abnormal lung imaging (%)	49(90.7)
Disease type (%)	
AML	27(50%)
ALL	14(25.9%)
MDS	6(11.1%)
AA	3(5.6%)
CMML	4(7.4%)
HSCT type (%)	
MAC-Haplo-HSCT	49(90.7%)
RIC-Haplo-HSCT	3(5.6%)
MAC-MSD-HSCT	2(3.7%)

*CRP* C-reactive protein, *IL-6* Interleukin-6, *PCT* Procalcitonin,* WBC* White blood cell, *HSCT* Hematopoietic stem cell transplantation, *MAC-Haplo-HSCT* Myeloablative Haploidentical hematopoietic stem cell transplantation, *RIC-Haplo-HSCT* Reduced-intensity conditioning haploidentical hematopoietic stem cell transplantation, *MAC-MSD-HSCT* Myeloablative matched-sibling donors hematopoietic stem cell transplantation, *allo-HSCT* Allogeneic hematopoietic stem cell transplantation, *ALL* Acute lymphoblastic leukemia, *CMML* Chronic myelomonocytic leukemia, *MDS* Myelodysplastic syndrome, *AA* Aplastic anemia

### Performance comparison of mNGS and CMT

**Fig. 1:**
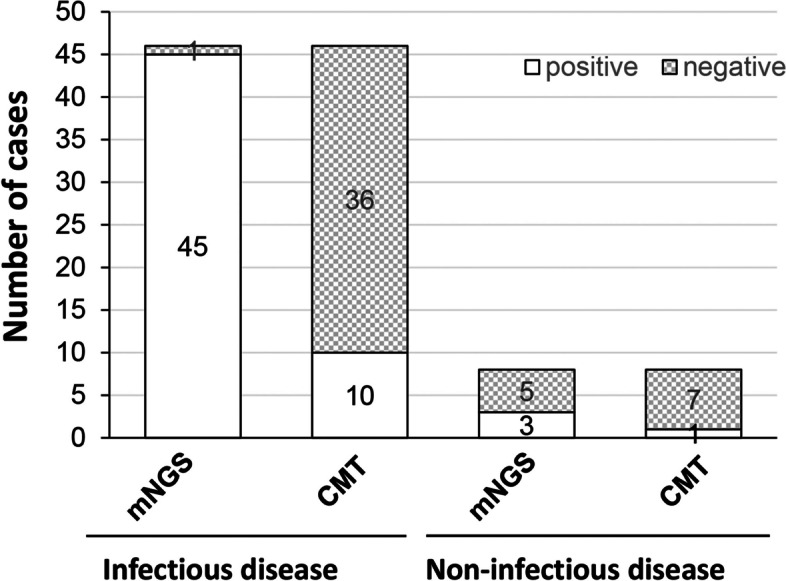
Comparison between mNGS and conventional microbiological tests (CMTs) in patients with infectious and non-infectious diseases

The positive rates of mNGS and CMT were compared (Fig. 1). The positive detection rate of mNGS was 88.9%, which was significantly higher than that of CMT (33.3%) (*P* < 0.001). Among the patients with infectious diseases (*n* = 46), 45 were detected positive by mNGS and 10 were detected positive by CMTs. The positive rate between mNGS and CMTs was significantly different (*P* < 0.05). For patients with non-infectious diseases (*n* = 8), 3 were detected positive by mNGS and 1 was detected positive by CMTs. For mNGS, the sensitivity was 97.82%, the specificity was 25%, the positive predictive value was 93.75%, and the negative predictive value was 50%. For CMTs, the sensitivity was 21.73%, the specificity was 100%, the positive predictive value was 100%, and the negative predictive value was 10%. The mNGS had significantly higher sensitivity but significantly lower specificity than CMTs (*P* < 0.05). Kappa-test showed that the agreement (40.74%) between mNGS and CMTs was good, with a kappa value of 0.059 (Table 2).

**Table 2:** Agreement between mNGS and CMT

	mNGS-positive	mNGS-negative	Total	Kappa, agreement
CMT-positive	17	1	18	0.059, 40.74%
CMT-negative	31	5	36	
Total	48	6	54	

*mNGS* Metagenomic next-generation sequencing, *CMT* Conventional microbiological test

### Analysis of identified pathogens

**Fig. 2:**
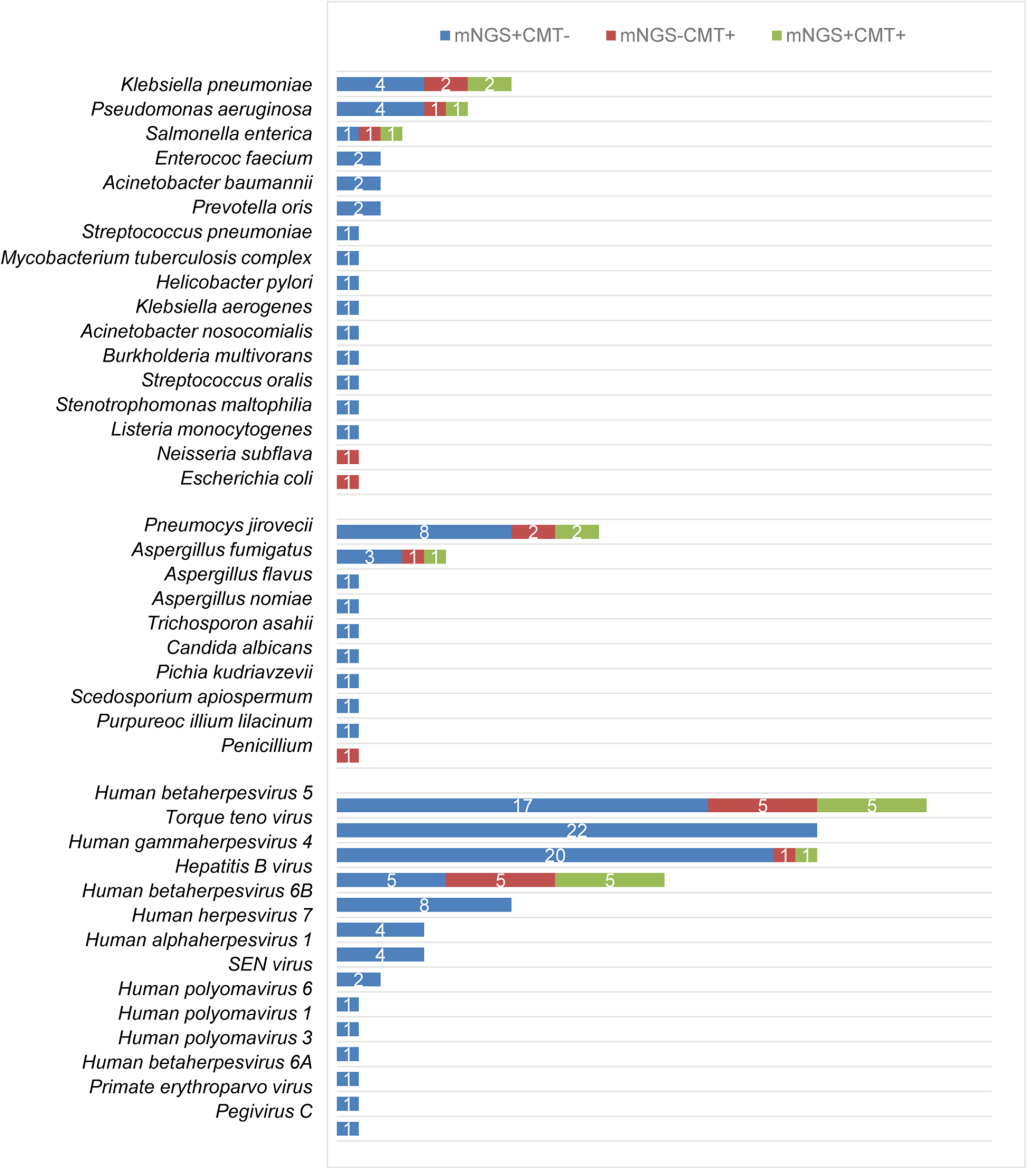
The information of 41 pathogens identified by mNGS and CMTs

A total of 41 pathogens were identified by both mNGS and CMTs (Fig. 2). Among them, 38 pathogens were identified by mNGS alone, whereas only 3 pathogens were identified by CMTs alone. There were 18 times matched results (18/41, 43.9%) between mNGS and CMTs, of which *Salmonella enterica* (*n* = 1 time) and hepatitis B virus (*n* = 5 times) were completely matched. There 12 times partially matched results between mNGS and CMTs, including *Klebsiella pneumoniae* (*n* = 2 times), *Pseudomonas aeruginosa* (*n* = 1 time), *Pneumocystis jirovecii* (*n* = 2 times), *Aspergillus fumigatus* (*n* = 1 time), EBV (*n* = 1 time), cytomegalovirus (CMV) (*n* = 5 times).

The common bacteria in patients after HSCT are *Klebsiella pneumoniae* and *Pseudomonas aeruginosa*. The common fungi are *Pneumocystis jirovecii* and *Aspergillus fumigatus*, and the common viruses are TTV, EBV, CMV, and human herpesvirus 6B (HHV-6B). In this study, we found that viruses were the most common pathogens in patients after HSCT, especially TTV.

**Fig. 3:**
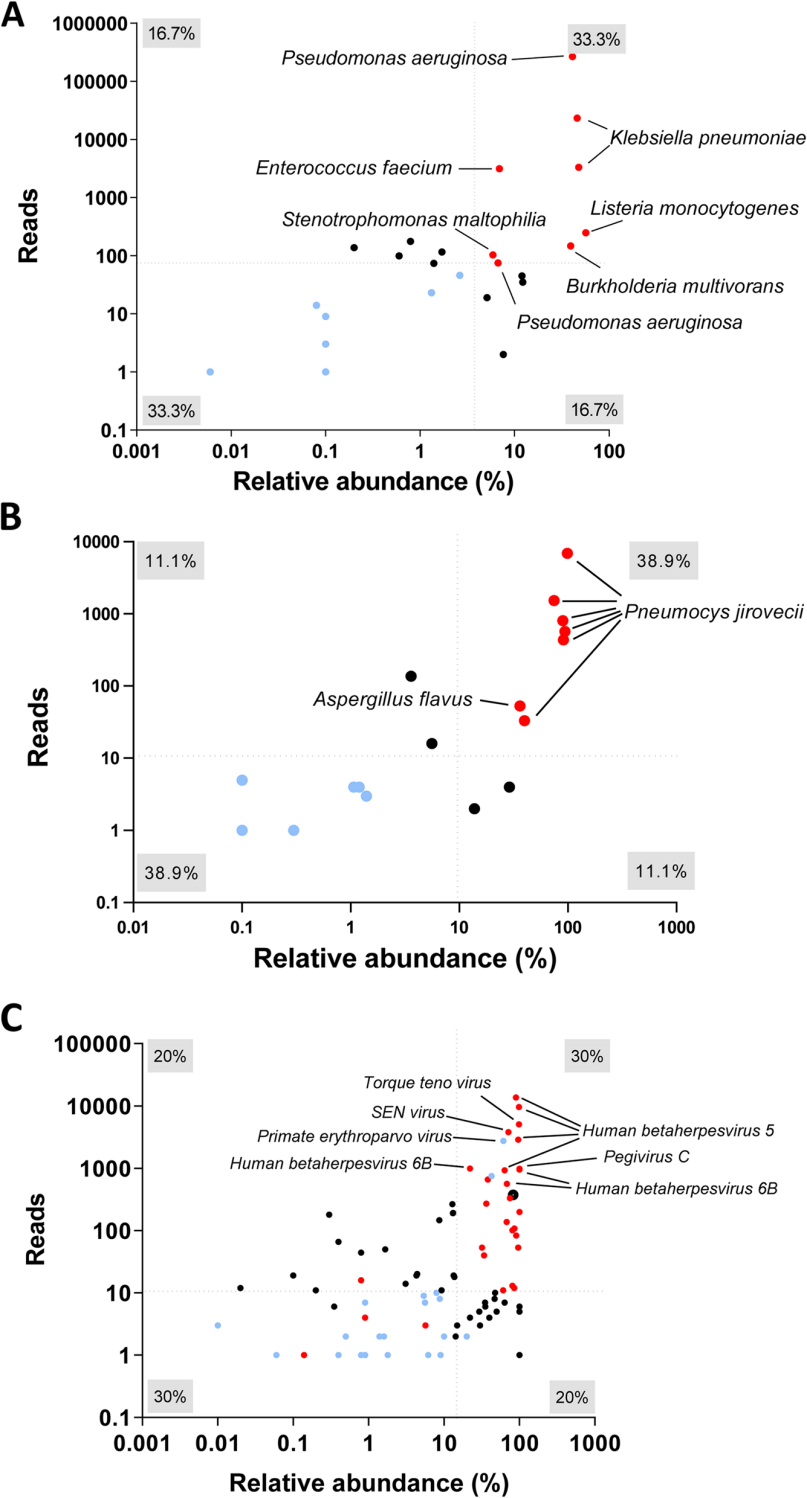
Scatter plot showing the distribution of 41 pathogens in patients after hematopoietic stem cell transplantation. Red dots represent potential pathogens, black dots represent other potential pathogens, and blue dots represent non-pathogens. Each quadrant’s percentage signifies the relative proportion of pathogenic microorganisms present in that quadrant. **A** Bacterial infection. Out of the bacterial genera, 33.3% met the outlier criteria when their reads were greater than 74.5 and relative abundance exceeded 3.8%. **B** Fungal infection. Among the fungal genera, 38.9% met the outlier criteria when their reads were greater than 10.5 and their relative abundance surpassed 9.6%. **C** Viral infection. In the case of viruses, 30% met the outlier criteria when their reads were greater than 10.5 and their relative abundance exceeded 14.65%

We plotted scatter plots based on sequencing reading and the relative abundance of pathogens. As shown in Fig. 3A, a total of 17 types of bacteria were detected, with a total of 24 bacterial instances. The bacterial species exhibited a sequence count ranging from 1 to 265,884 (with a median of 74.5), and their relative abundance varied from 0.1 to 56.78 (with a median of 3.8%). Among them, 33.3% (9 instances) of the bacterial genera were found to meet the outlier criteria (the median of the detected pathogens), classifying them as bacterial-positive. The dominant bacteria detected by mNGS included *Klebsiella pneumoniae* (*n* = 2), *Pseudomonas aeruginosa* (*n* = 2), *Enterococcus faecium* (*n* = 1), *Salmonella enterica* (*n* = 1), *Listeria monocytogenes* (*n* = 1), *Maltophila stenophila* (*n* = 1), and *Burkholderia multiphage* (*n* = 1). A total of nine different fungal species were detected. In total, 18 instances of fungal detection were recorded. The number of sequences per fungus ranged between 1 and 6926, with a median value of 10.5 sequences. The relative abundance ranged between 0.1% and 98.9%, with a median value of 9.6%. Additionally, 38.9% (7 instances) of the fungal genera met the outlier criteria, indicating that they were positively identified and categorized as fungi. The dominant fungi detected by mNGS included *Pneumocystis jirovecii* (*n* = 6) and *Aspergillus flavus* (*n* = 1) (Fig. 3B). There were 2 patients with infection of more than 1 fungus. A total of 14 types of viruses were detected, with a total of 90 instances of fungal detection. The number of virus sequences ranged from 1 to 13,652 (median 10.5 sequences), with relative abundance ranging from 0.1 to 100% (median 14.65%). 30% (27 instances) of the viruses met the outlier criteria, indicating identification and classification as potentially pathogenic viruses. The dominant virus was CMV (*n* = 5), followed by HHV-6B (*n* = 3), EBV (*n* = 2), and B19 virus (*n* = 1) (Fig. 3C).

### Factors associated with the results of mNGS

In this study, 61 mNGS tests were performed on 54 patients. Among them, 5 peripheral blood samples and 2 CSF samples were re-tested after treatment. The time intervals between testing varied from 8 ~ 72 days, and 1 ~ 4 different pathogens were detected each time. The pathogens were not completely consistent each time. Two patients died from recurrent infection with fungi and drug-resistant bacteria after transplantation (Table 3). One patient was infected with HHV-6B and CMV. In mNGS, although the number of HHV-6B sequences was only 7, the relative abundance reached 63.6%. Combined with the patient’s medical history, symptoms, and signs, this case was diagnosed as an HHV-6B viral infection. The mNGS was repeated after 14 days of treatment, revealing a negative HHV-6B. Although most of the detected pathogens were viruses, they were not the direct cause of death. Patients with non-resistant bacterial infections showed a favorable prognosis after receiving targeted treatment (Table 3).

**Table 3:** The mNGS and CMTs results of seven patients with repeated testing

Patient no.	Interval testing time for mNGS (Day)	Samples	mNGS	No. of reads	CMT	Blood culture	Clinical diagnosis	Outcome
26	15	Blood	TTV	199	Negative	Negative		Survival
		BALF	EBV TTV HHV-6B	2001 67 80	EBV-DNA	Negative	EBV	Survival
34	72	Blood	*Klebsiella pneumoniae* TTV	137 18	Negative	Neisseria flavescens	Bacteremia	Survival
		Blood	*Aspergillus terreus* *Aspergillus nidulans* *Aspergillus fumigatus* CMV	316 309 212 16	Negative	Negative	Pulmonary fungal infection	Died
36	14	Blood	HHV-6B CMV	7 1	Negative	Negative	HHV-6B Viral rash	Survival
		Blood	CMV	5	Negative	Negative		Survival
44	8	Blood	*Klebsiella pneumoniae* HHV-6B EBV	74 4 4	Negative	Negative	Bacteremia	Survival
		Blood	HHV-6B EBV CMV	409 12 11	Negative	Staphylococcus epidermidis	Staphylococcus epidermidis bacteremia	Died
46	0	Cerebrospinal fluid	*Listeria monocytogenes*	247	HBV-DNA (Blood)	Negative	Listeria monocytogenes meningitis	Survival
		Cerebrospinal fluid	Negative	Negative	HBV-DNA (Blood)	Negative		
48	10	Blood	*Prevotella oris* CMV	9 9623	CMV-DNA	Negative	CMV Viremia	Survival
		Blood	*Pseudomonas aeruginosa* CMV TTV EBV	27,780 112,298 5 4	Negative	Negative	Pseudomonas aeruginosa	Survival
49	0	Cerebrospinal fluid	HHV-6B	950	Negative	Negative	HHV-6B encephalitis	Survival
		Cerebrospinal fluid	Negative	Negative	Negative	Negative		

*mNGS* Metagenomic next-generation sequencing, *CMT* Conventional microbiological test, *TTV* Torque teno virus, *EBV* Epstein-Barr virus, *HSV-1* Human alpha herpes virus 1, *HPV-3* Human polyomavirus 3, *HHV-7* Human betaherpe svirus 7, *BALF* Bronchoalveolar lavage fluid, *CMV* Cytomegalovirus, *IP* Idiopathic pneumonia, *BOS* Bronchiolitis obliterans syndrome

For different samples, more viruses were detected from BALF than from blood samples in one patient (Table 3). Two patients were tested for CSF and blood samples simultaneously. Of note, the Listeria monocytogenes was detected in the CSF of one patient, and the remaining pathogens except Listeria monocytogenes in blood samples were exactly matched. The sequence number of the hepatitis B virus in blood samples was 679 times that in CSF. In the other patient, HHV-6B was detected in CSF, but not in blood samples. After treatment, mNGS of HHV-6B in CSF was negative (Table 3). mNGS offers comprehensive pathogen testing, but occasionally, it may detect results that are not directly linked to the patient’s clinical presentation. Therefore, it is essential to consider clinical symptoms, laboratory findings, and prior medical history when interpreting mNGS results.

### Treatment and prognosis

Among 46 cases of patients with infectious diseases diagnosed by mNGS, 43 patients modified their treatment plans and experienced positive outcomes. Three cases resulted in fatalities due to severe bacterial infections, specifically bloodstream infections. Two cases were attributed to *Klebsiella pneumoniae*, while the remaining case was caused by drug-resistant *Pseudomonas aeruginosa*. The treatment for bacterial infections showed poor efficacy, directly leading to death in these cases. In contrast, all eight patients with non-infectious diseases achieved positive therapeutic outcomes following targeted treatment.

## Discussion

In this study, 46 patients were diagnosed with infectious diseases. *Pneumocystis jirovecii* pneumonia (PJP) was first identified in our center by mNGS testing of BALF and imaging analysis. HHV-6B infection and *Listeria monocytogenes* infection were also first identified in our center by mNGS testing of CSF and the clinical manifestations. Additionally, *Aspergillus* infection was also first identified in our center by non-lung tissue biopsy, mNGS testing of blood samples, and imaging changes. Therefore, mNGS can be used to identify atypical organisms and diagnose clinically difficult cases [22].

The causative pathogens of neurological infectious complications after allo-HSCT are challenging to identify using CMTs and imaging. CSF is usually sterile and has a small cell count, which makes it difficult to simultaneously detect possible RNA viruses, DNA viruses, bacterial, fungal, and parasitic infections by CMT. Over 50% of the patients with suspected meningitis remain undiagnosed despite undergoing repeated CMTs, and a definite diagnosis needs to be confirmed by mNGS and other tests [23, 24]. The mNGS has advantages in diagnosing chronic meningitis and encephalitis. Carbo et al. [25] showed that in 18 immunocompromised patients, the pathogens were identified by mNGS analysis of CSF. In this study, the pathogens of *Listeria monocytogenes* and HHV-6B were detected by CSF mNGS test in two cases with negative CSF CMT results. After targeted treatment, the CSF mNGS retest was negative and had 100% sensitivity and specificity. Neurological infectious complications are important when symptoms such as headache, memory loss, confusion, and seizures occur without obvious cause after allo-HSCT, and the CSF mNGS test is important to confirm the clinical diagnosis. Miller et al. [26] developed and validated a clinical mNGS assay for the diagnosis of infectious causes of meningitis and encephalitis from CSF in a licensed microbiology laboratory. A customized bioinformatics pipeline, SURPI+, was developed to rapidly analyze mNGS data, generate an automated summary of detected pathogens, and provide a graphical user interface for evaluating and interpreting results. With technological advancements, the interpretation of mNGS results is expected to further improve.

In this study, the sequence number of bacteria by mNGS was higher than that of fungi and viruses, but the relative abundance was lower than that of most fungi and viruses. In 18 patients, 20 bacteria were found, including *Pseudomonas aeruginosa*, *Enterococcus fecal*, *Acinetobacter baumannii*, *Klebsiella pneumoniae*, *Burkholderia polyphagocytosis*, etc. The mNGS results of 9 patients met the outlier criteria, and they were diagnosed as bacterial infections combined with a comprehensive analysis of the clinical situation. Among them, 3 patients died due to carbapenem-resistant *Enterobacteriaceae*, 2 cases of *Klebsiella pneumoniae*, and 1 case of *Pseudomonas aeruginosa* within 1 month after haploid transplantation. Clinical symptoms of infection with multidrug-resistant bacteria are more complex and severe than nondrug-resistant bacterial infections and pose serious challenges to treatment [25, 27]. For patients with fever in the nonfunctional phase of bone marrow after transplantation, mNGS testing may detect carbapenem-resistant *Enterobacteriaceae* in a more timely and accurate manner than CMT, thereby improving efficacy. Zhang et al. [28] detected *Listeria monocytogenes* infection by CSF mNGS and the patient was improved after treatment. In this study, one patient with persistent fever, headache, nausea, and vomiting was confirmed to have *Listeria monocytogenes* infection by CSF mNGS and recovered after treatment. We also found that almost all patients with blood infections after allo-HSCT were detected to have more than 2 pathogenic bacteria in blood samples. The blood infections were mostly bacterial combined with viral infections, some were bacterial combined with fungal infections, and some were even bacterial, viral, and fungal infections, which may be related to the low immune function of patients following allo-HSCT. The multiple infections can affect each other and may lead to complex clinical manifestations. The diversity of infections also partly explains the failure of empiric antibacterial treatments. CMTs cannot simultaneously detect multiple pathogens. mNGS can simultaneously detect specific pathogens and can identify a wide range of different types of pathogens [22] and has been less commonly affected by previous history of antibiotics and glucocorticoid administration [29]. Therefore, mNGS is more suitable for detecting pathogens in critically ill patients, such as patients with immunodeficiency after allo-HSCT. In this study, the identified pathogens by mNGS coincided with the clinical diagnosis of patients, and the success rate of antibacterial infection treatment guided by mNGS results was higher.

Diagnosis of fungal infections of the lungs after allo-HSCT is challenging. Hong et al. [14] showed that fungal infection in patients was diagnosed with mNGS analysis of plasma specimens and was validated by biopsy. However, in clinical practice, there is a certain risk for lung biopsy. Chellapandian et al. [30] conducted a mating analysis of 14,148 studies and found that lung biopsies in patients with HSCT resulted in a four-fold increase in mortality. Therefore, mNGS of plasma and BALF are considered a “liquid biopsy” of diagnostic value [31].

In this study, mNGS testing of plasma and BALF also improved the clinical diagnosis of invasive fungal infections in the lungs. PJP was the most common fungal infection after allo-HSCT in this study, and all were identified from BALF specimens and by mNGS, thus avoiding the risks associated with lung biopsy. Wang et al. [32] showed that the sensitivity and specificity for diagnosing PJP by mNGS were 100% and 91%, respectively, and that the specimen source was preferably BALF, followed by whole blood. After transplantation, patients have low immune function, the prevention of PJP is very important, and, sufficient doses and courses of sulfamethoxazole-trimethoprim are needed. In this study, one patient was diagnosed with *Aspergillus flavus* infection through peripheral blood mNGS. For hard-to-culture fungi (e.g., *Aspergillus*, *Pneumocystis jeroveci*, etc.), direct sequencing of plasma specimens provides a faster diagnosis and avoids the risks associated with invasive biopsies [32]. Langelier et al. [33] showed that at least one pathogen detected by CMT was identified by plasma mNGS in 67% of cases, and pathogens were confirmed by culture in 44% of cases, while mNGS testing substantially increased the pathogen diagnosis rate in lower respiratory tract samples to 100% and 61%, respectively. Therefore, for post-transplant patients with lung imaging changes, it is recommended to perform bronchoscopy and BALF mNGS in time, to improve the diagnosis rate of pulmonary fungal infection. For patients who have difficulty in receiving bronchoscopy, mNGS analysis of the plasma and blood specimens can improve the diagnosis rate of fungal infection. Timely diagnosis of fungal infections could help guide treatment, avoiding excessive use of empiric antimicrobial drugs, and improving genetic testing of fungal resistance.

Virus infection after allo-HSCT is complex and has diverse presentations, which may cause misdiagnosis or missed diagnosis. Opportunistic viral infections often occur after solid organ transplantation or HSCT, and the associated morbidity and mortality rates are as high as 40% [25]. Zanella et al. [34] found that TTV was more common in patients with GVHD treated with corticosteroids, and concluded that viremia was not significantly related to clinical manifestations. In this study, TTV was the most common virus, followed by EBV, CMV, and HHV-6B. However, TTV infection is not considered to be the cause of virus-associated pneumonia and is more considered a potential pathogen in immunocompromised patients [35]. In this study, there was no CMV-related disease, but the patients had CMV-DNA aemia. Five patients were presented with fever and gradually declined platelets, and confirmed to have CMV infection by plasma mNGS. CMV-DNA was only detected by CMT (copy number > 5.00E + 02IU / ml) until about 1 week later. It is shown that virus replication can be detected by CMT in the setting of proliferative virus infection [36], which is consistent with the results of this study, suggesting that mNGS of plasma and blood samples can achieve early diagnosis of CMV and other viral infections. Combined with the clinical manifestations of patients, CMV prevention can be implemented. Meanwhile, it is necessary to dynamically monitor the virus copy in immunocompromised patients after allo-HSCT, and when the copy number is not less than 5.00E + 02IU / ml, special attention should be paid. For EBV, when the DNA copy number is not less than 5.00E + 02IU/ml, plasma mNGS, especially mNGS of blood samples, is particularly important. However, more cases are required to confirm this result.

In this study, CMV and EBV were detected by mNGS in one patient with BOS and one patient with IP, respectively. Zhou et al. [37] found that the HHV-6B or EBV infection within 100 days of transplantation increased the risk of IP, and the CMV infection increased the risk of BOS and grade II–IV GVHD. Future monitoring of CMV and EBV infection in patients with BOS and IP is necessary. HHV-6B infection can manifest as viral encephalitis, rash, etc., which can be inherited from parents to children, and be transmitted from donors to transplanted patients. HHV-6B is also one of the causes of myelosuppression and allogeneic transplant failure. HHV-6 measured in plasma and CSF is considered infectious [38, 39]. In this study, 1 patient with persistent fever, pancytopenia, and a generalized rash was identified to have HHV-6B infection by plasma mNGS, but the CMT result was negative. One patient with fever, epilepsy, and disturbance of consciousness was diagnosed with viral meningitis caused by HHV-6B by CSF mNGS. At present, PCR is still a commonly used method for detecting viruses, but mNGS can detect a wider range and has advantages in the clinical diagnosis of viral infection after allo-HSCT [40]. In addition, mNGS of peripheral blood samples is very important to rule out infectious diseases in patients with engraftment syndrome and fever caused by GVHD after allo-HSCT.

False positive results may be generated due to the high sensitivity of mNGS. The BALF and CSF specimens do not require liquefaction treatment, reducing the chance of contamination, and thus resulting in a high positive detection rate. Different kinds of antibiotics would be administered in patients with allo-HSCT. Compared with CMT, mNGS tests are not affected by the application of antibiotics, but it should be noted that with the extension of anti-infection treatment time, the detection rate of mNGS on pathogenic microorganisms gradually decreases, early mNGS testing is required depending on the patient’s condition [41]. The application of mNGS in clinical practice is becoming more and more mature and extensive, and it has been recommended as the first-line test to confirm the clinical diagnosis of pathogens in patients with allo-HSCT [42]. In this study, we also showed that although blood cultures in patients with pneumonia were negative, pathogen DNA could also be found in the blood. Although BALF mNGS is more sensitive than blood mNGS in detecting bacteria and fungi, blood mNGS still has a certain value in the clinical diagnosis of lung infections, especially in patients with viral and fungal infections when respiratory specimens are not available.

It is critical to analyze the mNGS results more effectively. Based on our limited experience, it is not sufficient to only rely on reads to identify pathogens. Reads to pathogenic bacteria that are not usually colonizing bacteria support the diagnosis of an infection. In particular, pathogens known to cause immune system infections, such as *mycobacterium tuberculosis*, *cryptococcus*, *legionella pneumophila*, *parrot chlamydi*a, and *parasites* (such as *toxoplasma*), do not necessarily adhere to colonization patterns and should be carefully considered as potentially infectious agents in specimens detected by mNGS. Bacteria and fungi identified through mNGS should be evaluated for their pathogenic potential based on local infection epidemiology, infection site, and the patient’s colonization patterns. A high read detection of a single bacterium or fungus in samples often indicates pathogenic microorganisms. However, when detecting multiple microorganisms, their interpretation should consider the common colonization sites of the detected pathogens and the possible route of entry of their nucleic acids into the blood.

### Study limitations

There were several limitations in this study. Firstly, there was a relatively small sample size, which limited the power of confirmation of the diagnostic value of mNGS in infectious diseases in patients receiving allo-HSCT. Secondly, the optimal timing for the application of mNGS in patients after allo-HSCT has not yet been determined. Thirdly, secondary testing was not performed to confirm the initial identification by mNGS. Finally, only DNA was sequenced by mNGS and any RNA viruses would likely not be detected through this method. Further studies with larger sample sizes are needed.

## Conclusions

The mNGS has emerged as a crucial detection method in the context of infections following allo-HSCT. This method is not affected by immunosuppression status, empiric antibiotic therapy, and multi-microbial mixed infection. However, our experience has revealed that mNGS detection also comes with limitations. First, in suspected cases of catheter-related bloodstream infections, mNGS is not the preferred testing method. Moreover, the diagnostic efficacy of blood mNGS for lower respiratory tract infections is limited. Furthermore, it is important to note that the results of mNGS detection rely heavily on the quality of the specimen. During specimen collection and transportation, strict adherence to aseptic procedures and prompt transportation principles is essential. Additionally, although virus detection is common in mNGS reports of hematological patients, it does not necessarily indicate virus replication. PCR testing should be utilized to evaluate the viral load. In determining the nature of the microorganisms detected by mNGS—whether they signify an infection, colonization, or contamination—it is essential to consider the patient’s clinical manifestations, imaging features, and other test results.

## Data Availability

The datasets used and/or analyzed during the current study are available from the corresponding author upon reasonable request.

## Abbreviations

mNGS: Metagenomic next-generation sequencing
allo-HSCT: Allogeneic hematopoietic stem cell transplantation
CMT: Sconventional microbiological tests
GVHD: Graft-versus-host disease
CSF: Cerebrospinal fluid
BALF: Bronchoalveolar lavage fluid
BOS: Bronchiolitis obliterans
IP: Interstitial pneumonia
